# Cancer Prevention and Health Benefices of Traditionally Consumed *Borago officinalis* Plants

**DOI:** 10.3390/nu8010048

**Published:** 2016-01-18

**Authors:** María-Dolores Lozano-Baena, Inmaculada Tasset, Andrés Muñoz-Serrano, Ángeles Alonso-Moraga, Antonio de Haro-Bailón

**Affiliations:** 1 Department of Plant Breeding, Institute of Sustainable Agriculture, CSIC, Av. Menéndez Pidal s/n, Córdoba E-14004, Spain; adeharobailon@ias.csic.es; 2 Department of Developmental and Molecular Biology, Institute for Aging Studies, Albert Einstein College of Medicine, 1300 Morris Park Avenue, Bronx, NY 10461, USA; inmaculada.tasset@einstein.yu.edu; 3 Department of Genetics, Gregor Mendel Building, Faculty of Science, University of Córdoba, Campus Rabanales, Córdoba 14014, Spain; ge1ams@uco.es (A.M.-S.); ge1almoa@uco.es (Á.A.-M.)

**Keywords:** *Borago officinalis*, health, safety, dietary bioactives, vegetables, SMART, HL-60, cancer prevention

## Abstract

Nowadays, healthy eating is increasing the demand of functional foods by societies as sources of bioactive products with healthy qualities. For this reason, we tested the safety of the consumption of *Borago officinalis* L. and its main phenolic components as well as the possibility of its use as a nutraceutical plant to help in cancer prevention. The *in vivo Drosophila* Somatic Mutation and Recombination Test *(*SMART) and *in vitro* HL-60 human cell systems were performed, as well-recognized methods for testing genotoxicity/cytotoxicity of bioactive compounds and plant products. *B. officinalis* and the tested compounds possess antigenotoxic activity. Moreover, *B. officinalis* wild type cultivar exerts the most antigenotoxic values. Cytotoxic effect was probed for both cultivars with IC_50_ values of 0.49 and 0.28 mg·mL^−1^ for wild type and cultivated plants respectively, as well as their constituent rosmarinic acid and the assayed phenolic mixture (IC_50_ = 0.07 and 0.04 mM respectively). *B. officinalis* exerts DNA protection and anticarcinogenic effects as do its component rosmarinic acid and the mixture of the main phenolics presented in the plant. In conclusion, the results showed that *B. officinalis* may represent a high value plant for pleiotropic uses and support its consumption as a nutraceutical plant.

## 1. Introduction

Healthy eating is one of the most pursued objectives in today’s society. The increased demand for food with protection properties against diseases has made herbal products a principal target for industry requirements and government recommendations. In this sense, people usually search for plants according to their well recognized benefits for human health, and most commonly herbal components are considered commercial products [1]. However, reports that show protective effects in some species are often conflicting or present variable results.

Borage (*Borago officinalis* L., *Boraginaceae*), also known as starflower, is a native annual plant in the Mediterranean region that has been used since ancient times for culinary and medicinal purposes, for the treatment of swelling and inflammation, respiratory complaints and melancholy (“I, Borage, bring always courage,” translation of the old verse “*Ego borago gaudia semper ago*”) [2]. Also, health properties such as anti-obesity, diuretic, emollient, lenitive, laxative, anti-anemic, menstrual analgesic and antipyretic properties are recorded [3,4,5]. In this sense, borage leaves (>60% of the plant matter) are considered by industries as a by-product, so it could be used as an economic source of healthy products [6].

Vegetable use of borage is common in Germany (as an ingredient in green sauce, made in Frankfurt), Crete and in the Italian region of Liguria (to fill traditional ravioli pasta). Vegetable borage is also very popular in the cuisine of the Spanish regions of Aragon and Navarra (*i.e.*, boiled and sautéed with garlic, served with potatoes). Borage is used by naturopathic practitioners in the regulation of metabolism and the hormonal system, being considered a good remedy for premenstrual syndrome and menopause symptoms, such as hot flashes [7,8]. In Iran, people make tea (Gol Gav Zaban tea) to relieve colds, flu, bronchitis, rheumatoid arthritis, and kidney inflammation [9]. Recently, interest in borage has been renewed because its seeds appear to be the richest known plant source of gamma linolenic (all *cis*-6,9,12 octadecatrienoic) acid (GLA), which is an intermediate of indispensable compounds in the body, such as prostaglandin E1 and its derivatives [10,11,12,13,14]. All these facts have generated an increasing interest in *B. officinalis* production and researchers are now establishing the best management practices in order to optimize crop performance [15,16]. Furthermore, borage is used by industries as an antioxidant due to its bioactive compound content, *i.e.*, phenolics, responsible for most plant properties [17,18,19]. The phenolic content of edible parts (leaves and petioles) of *B. officinalis* had been previously determined, with rosmarinic, syringic and sinapic acids being the major phenolics in all plant growth stages [20,21,22]. These three compounds act as bioactive molecules and exert antioxidant and anti-inflammatory properties [23,24,25]. Specially, rosmarinic acid is investigated and employed by the food and pharmaceutical industries [26].

The complexity of plant composition and the human digestion process requires validated models that represent this relation as closely and in a manner as valuable for research as possible. For this reason, we have selected the *in vivo Drosophila melanogaster* and *in vitro* HL-60 human cancer cell system as two complementary, sensitive, low-cost and rapid eukaryotic assays, able to detect the potential mutagenic and carcinogenic effects of tested compounds [27,28,29,30].

We present the first report proving the antigenotoxic and anticarcinogenic properties of two *B. officinalis* varieties (wild and cultivated) as well as of their major phenolics: rosmarinic, syringic and sinapic acids. Moreover, the interaction between these bioactive compounds is tested, highlighting their potential use and commercialization by industries for products with health benefits as dietary bioactives.

## 2. Materials and Methods

### 2.1. Plant Material

Two *Borago officinalis* L. varieties were selected for this work: blue-flowered (BF, wild type, accession Bo IAS 2008-07, collected in Córdoba in December 2009, Southern Spain) and white-flowered (WF type, accession Bo IAS 2008-08, traditionally cultivated in Navarra in December 2009, Northern Spain). These genotypes are part of a *B. officinalis* germplasm bank in the Institute of Sustainable Agriculture (IAS-CSIC, Córdoba, Spain). Plants were grown on an experimental farm at the IAS (N 37°8', W 4°8') wherein climate is typically Mediterranean, with an average annual rainfall of 650 mm. The soil is deep and sandy-loam, classified as a Typic Xerofluvent. Leaves and petioles from 5 plants of each variety were harvested when they reached the optimal stage to be consumed (55 days after sowing), weighed, frozen (24 h at −80 °C) and lyophilized with a freeze-drier Telstar model Cryodos-50 (Telstar, Terrasa, Spain). After lyophilisation, dry material was weighed again, grounded for about 20 s in a Janke and Kunkel Model A10 mill (IKA-Labortechnik, Staufen, Germany), mixed and kept at room temperature and in darkness to preserve their properties until use.

### 2.2. Chemicals

The single compounds, rosmarinic (C_18_H_16_O_8_), syringic (C_19_H_10_O_5_) and sinapic (C_11_H_12_O_5_) acids, were purchased from Sigma-Aldrich (St. Louis, MO, USA).

### 2.3. Drosophila Experiments

#### 2.3.1. Fly Stocks and Crosses

The *D. melanogaster* system was selected for the determination of the safety of *B. officinalis* consumption as a well-recognized method to analyze vegetable complex mixtures using SMART [31,32]. This test was used in order to evaluate the genotoxic and antigenotoxic activity of *B. officinalis* leaves and petioles as well as their selected bioactive compounds [33]. This activity was measured by direct visualization of the occurrence of recessive mutations in the wing hairs of two different *D. melanogaster* strains. Flies from experiments carried these visible wing genetic markers: the flare (*flr*) *s*train *flr*^3^/*ln (3LR) TM3*, *Bd^s^* [34] and the multiple wing-hair (*mwh*) strain *mwh*/*mwh* [35]. The marker flare (*flr*^3^, *3_38.3*) produces individual malformed hairs and the marker multiple wing hairs (*mwh*, *3*_*0.3*) produces multiple hairs per cell. Larvae used in treatments come from two types of crosses: the standard cross with *flr*^3^/*TM*3*, Bd^S^* females mated to *mwh*/*mwh* males and the reciprocal cross.

#### 2.3.2. Larvae Treatments

Optimal fertile flies were anesthetized under CO_2_ narcotisation for cross selection and then placed in new vials for fertilization. After that, hybrid eggs from crossing were collected over an 8 h period and emerged larvae (72 ± 4 h later) were cleaned up for a few seconds in sterile distilled water to remove feeding medium rests [33]. For genotoxicity analysis (simple treatments), groups of 100 larvae were transferred into vials containing 0.85 g of *Drosophila* Instant Medium (Formula 4–24, Carolina Biological Supply, Burlington, NC, USA) wetted with 4 mL of a mixture of distilled water and increasing concentrations of samples: *B. officinalis* BF and WF (1.25, 2.5, and 5 mg·mL^−1^), RO (0.35, 0.7, 1.39 and 2.78 mM), SY (0.16, 0.32, 0.63 and 1.26 mM), SI (0.15, 0.29, 0.58 and 1.16 mM) and the mixture of these three bioactive compounds at each concentration assayed individually. Bioactive compound concentrations were chosen on the basis of their known content in *B. officinalis* species [11]. For antigenotoxicity analysis (combined treatments) the same number of vials were prepared but treatment media were mixed with H_2_O_2_ 0.12 M as mutagenic agent. Vials with the medium mixed with distilled water or H_2_O_2_ (0.12 M) were used as negative and positive controls respectively. Larvae were fed on these media until pupation (about 48 h). After emergence, resulting adult flies were sacrificed under CO_2_ narcotisation and stored in a 70% ethanol solution in sterile water. Emerged adults were counted for toxicity evaluation and transheterozygous wings (*mwh flr^+^/mwh^+^ flr*^3^) were mounted on microscope slides and wing hair mutations (spots) scored, using a photonic microscope (Nikon) at 400× magnification for genotoxicity and antigenotoxicity evaluation.

### 2.4. HL-60 Experiments

#### 2.4.1. Cell Cultures

The human acute promyelocytic leukemia cell line HL-60 was routinely grown in suspension in RPMI medium (Invitrogen, Madrid, Spain) containing glutamine (200 mM, Sigma-Aldrich, St. Louis, MO, USA), antibiotics (100IU penicillin mL^−1^ and 100 μg streptomycin mL^−1^, Sigma-Aldrich) and supplemented with 10% heat-inactivated foetal bovine serum (Linus, Cultek, Madrid, Spain) and placed in an incubator (Shel Lab, Cornelious, OR, USA) with a 5% CO_2_ humidified atmosphere at 37 °C [36]. HL-60 cells were subcultured every 2–3 days to maintain logarithmic growth and they were allowed to grow for 48 h before use [37]. Cultures were plated at a density of 12.5 × 10^4^ cells mL^−1^ in 40 mL culture flasks (25 cm^2^).

#### 2.4.2. Cell Treatments

Cytotoxic activity was measured as growing inhibition or decreased viability on HL-60 cells following a previous protocol modified by us [38]. For assays, cells were placed in 12-well culture plates (1 × 10^5^ cells mL^−1^; final volume = 2 mL per well) and treated with different filtered (Millipore “non-pyrogenic”, “sterile-R”, 0.2 μm filter) RPMI solutions with the selected concentrations of *B. officinalis* BF and WF plant samples (0.125, 0.25, 0.5, 1 and 2 mg·mL^−1^), RO (0.07, 0.14, 0.28, 0.55, 1.1 and 2.2 mM), SY (0.03, 0.06, 0.13, 0.25, 0.5 and, 1 mM), SI (0.03, 0.06, 0.12, 0.23, 0.5 and, 1 mM) and the mixture of these three bioactive compounds at each individually assayed concentration. Cells were counted after 72 h treatment. Tested concentrations were calculated according to those used for *in vivo* assays to equal the range of tested doses. Untreated cultures were used as negative control.

#### 2.4.3. Trypan Blue Dye Exclusion Assay

Cell viability was determined by the Trypan Blue dye exclusion test. Cells were stained with an equal volume of Trypan Blue commercial solution (Sigma-Aldrich) and counted using a hemocytometer at room temperature under a light inverted microscope (AE30/31, Motic, Barcelona, Spain).

### 2.5. Statistical Analysis

The determination of Toxicity (T) of treatments in *D. melanogaster* was performed following this formula [39]:

T = (N° of emerging individuals in treatment/N° of emerging individuals in the negative control) × 100(1)

Differences in *D. melanogaster* survival between treatments at each concentration with respect to negative control were analyzed with a Chi-square test. This procedure was also performed for the analysis of each simple treatment with their correspondent combined treatment.

For the evaluation of genotoxic effects, the frequencies of spots per fly of each treated series were compared to the concurrent negative control for each class of mutational clone as well as between simple and combined treatments for the same concentration comparisons. Spots were grouped into three different categories: single (a small single spot corresponding to one or two cells exhibiting the *mwh* phenotype), large (a large single spot corresponding to three or more cells showing *mwh* or *flr*^3^ phenotypes) and twin (a large spot corresponding to three or more cells showing adjacent both *mwh* and *flr*^3^ phenotypes). A multiple-decision procedure was used to categorize results as positive, inconclusive or negative [40]. Inconclusive and positive results were evaluated by the non-parametric *U* test of Mann, Whitney and Wilcoxon [41]. The inhibition percentage (IP) of genotoxicity was calculated from the total frequencies of spots per wing, following this formula [42]:

IP = (genotoxin alone − sample + genotoxin) × 100/(genotoxin alone)(2)

Significant differences of IP for each treatment respect to the positive control were analyzed with a Chi-square test.

Cytotoxic effect evaluation was determined after each culture incubation period, establishing a growth curve and determining IC_50_ values by regression analysis of the curves. Viability estimated regressions of leukemia cells are presented as a survival percentage with respect to controls at 72 h growth and plotted as mean viability ± standard error of at least three independent replicas for each treatment and concentration.

Statistical analyses were performed using a Microsoft 2007 Excel spreadsheet. The non-parametric U test of Mann, Whitney and Wilcoxon was performed with the SPSS Statistic 17.0 software (SPSS, Inc., Chicago, IL, USA).

## 3. Results and Discussion

### 3.1. *In Vivo* Assays

Table 1, Table 2 and Table 3 show the results obtained in *D. melanogaster* experiments for edible leaves and petioles of *B. officinalis* of the selected varieties, blue flowered (BF) and white flowered (WF), and their bioactive compounds, rosmarinic (RO), syringic (SY) and sinapic (SI) acids. The negative controls produced mutation rates which fall into the normal range obtained in other laboratories, thus the data in discussion comply with the expected spots per wing with no anomalous or borderline controls [43,44]. The positive control used in this study was hydrogen peroxide (H_2_O_2_). This oxidative mutagen has been used in many mutation assays and it is known that an excess of H_2_O_2_ can influence the expression of a high number of genes [45]. As previously reported, H_2_O_2_ affects *D. melanogaster* survival and creates an excess of small single spots, with no significant induction of twin spot excess [29,39]. The genotoxic results for H_2_O_2_ validate the assay as an appropriate system for screening between mutagens (positive controls as H_2_O_2_) and non-mutagens (water controls or safe plants and bioactive compounds).

**Table 1:** Toxicity of *Borago officinalis* plant material, blue flowered (BF) and white flowered (WF), and the bioactive compounds, rosmarinic (RO), syringic (SY) and sinapic (SI) acids.

Survival ^1^ % Treatments					
Simple		Combined ^2^	Simple		Combined ^2^
H_2_O	100		H_2_O_2_ (0.12 M)	37.87 *	
BF (mg·mL^−1^)			WF (mg·mL^−1^)		
1.25	100	52.44 *^,‡^	1.25	97.78	33.33 *^,‡^
2.5	100	54 *^,‡^	2.5	63.11 *	27.56 *^,‡^
5	82 *	86.89 *	5	71.33 *	17.33 *^,‡^
RO (mM)			SY (mM)		
0.35	48.44 *	49.56 *	0.16	39.78 *	31.11 *^,‡^
0.7	22.22 *	31.11 *^,‡^	0.32	42.67 *	29.33 *^,‡^
1.39	33.33 *	45.56 *^,‡^	0.63	31.11 *	20.44 *^,‡^
2.78	21.33 *	38.89 *^,‡^	1.26	58.22 *	36.89 *^,‡^
SI (mM)			RO + SY + SI (mM)		
0.15	78.22 *	64 *^,‡^	*a* ^3^	48.67 *	24.44 *^,‡^
0.29	60.22 *	58.89 *	*b*	55.11 *	34.67 *^,‡^
0.58	69.33 *	39.78 *^,‡^	*c*	74.44 *	57.78 *^,‡^
1.16	55.11 *	43.56 *^,‡^	*d*	44.89 *	53.78 *^,‡^

^1^ Survival expressed in percentage as total emerged adults of each treatment with respect to H_2_O control total emerged adults; ^2^ Combined treatments using standard medium and 0.12 M H_2_O_2_; ^3^ Letters *a*–*d* correspond to the lowest, two intermediate and highest concentrations respectively assayed for each single compound once their mixture is assayed; * Significance levels with respect to the negative control (untreated, H_2_O) group (*p* ≤ 0.05); ^‡^ Significance levels between simple and combined treatment for the same concentration comparisons (*p* ≤ 0.05).

**Table 2:** Genotoxicity of *Borago officinalis* plant material: blue flowered (BF) and white flowered (WF); and the bioactive compounds: rosmarinic (RO), syringic (SY) and sinapic (SI) acids.

Mutation Rate (Spots/Wing) Diagnosis ^1^					
	N° of Wings	Small Single Spots 1–2 Cells *m* = 2	Large Single Spots >2 Cells *m* = 5	Twin Spots *m* = 5	Total Spots *m* = 2
H_2_O	212	0.26 (54)	0.04 (8)	0.03 (5)	0.32 (67)
H_2_O_2_ (0.12 M)	168	0.60 (94) +	0.07 (11) −	0.06 (4) −	0.65 (109) +
BF (mg·mL^−1^)					
1.25	40	0.13 (5) −	0.03 (1) −	0.05 (2) −	0.20 (8) −
2.5	54	0.22 (12) −	0.06 (3) −	0.02 (1) −	0.30 (16) −
5	66	0.29 (19) −	0.03 (2) −	0.05 (3) −	0.36 (24) −
WF (mg·mL^−1^)					
1.25	66	0.26 (17) −	0.03 (2) −	0.05 (3) −	0.33 (22) −
2.5	50	0.26 (13) −	0.08 (4) −	0.02 (1) −	0.36 (18) −
5	90	0.36 (32) −	0.02 (2) −	0.01 (1) −	0.39 (35) −
RO (mM)					
0.35	16	0.38 (6) −	0	0	0.38 (6) −
0.7	34	0.21 (7) −	0	0.06 (2) −	0.26 (9) −
1.39	22	0.18 (4) −	0	0.05 (1) −	0.23 (5) −
2.78	38	0.16 (6) −	0.05 (2) −	0	0.21 (8) −
SY (mM)					
0.16	40	0.30 (12) −	0.05 (2) −	0.03 (1) −	0.38 (15) −
0.32	30	0.20 (6) −	0.07 (2) −	0	0.27 (8) −
0.63	48	0.19 (9) −	0.02 (1) −	0	0.21 (10) −
1.26	32	0.22 (7) −	0.06 (2) −	0	0.28 (9) −
SI (mM)					
0.15	24	0.38 (9) −	0.04 (1) −	0.04 (1) −	0.46 (11) −
0.29	32	0.39 (12) −	0.10 (3) −	0	0.48 (15) −
0.58	30	0.33 (10) −	0.07 (2) −	0	0.40 (12) −
1.16	40	0.23 (9) −	0.03 (1) −	0.03 (1) −	0.28 (11) −
RO + SY + SI (mM)					
*a* ^2^	26	0.15 (4) −	0	0.04 (1) −	0.19 (5) −
*b*	34	0.12 (4) −	0.03 (1) −	0	0.15 (5) −
*c*	32	0.22 (7) −	0.13 (4) +	0	0.34 (11) −
*d*	22	0.41 (9) −	0.05 (1) −	0	0.45 (10) −

**^1^** Statistical diagnoses: + (positive) and − (negative) [40,41]. Significance levels α = β = 0.05, one-sided test without Bonferroni correction; ^2^ Letters *a*–*d* correspond to the lowest, two intermediate and highest concentrations respectively assayed for each single compound once their mixture is assayed.

**Table 3:** Antigenotoxicity of *Borago officinalis* plant material: blue flowered (BF) and white flowered (WF); and the bioactive compounds: rosmarinic (RO), syringic (SY) and sinapic (SI) acids.

Mutation Rate (Spots/Wing) Diagnosis ^1^					
	N° of Wings	Small Single Spots 1–2 Cells *m* = 2	Large Single Spots >2 Cells *m* = 5	Twin Spots *m* = 5	Total Spots *m* = 2
H_2_O	212	0.26 (54)	0.04 (8)	0.03 (5)	0.32 (67)
H_2_O_2_ (0.12 M)	168	0.60 (94) +	0.07 (11) –	0.06 (4) –	0.65 (109) +
BF (mg·mL^−1^)					
1.25	30	0.13 (4) −	0.03 (1) −	0	0.17 (5) −
2.5	34	0.24 (8) −	0.03 (1) −	0	0.26 (9) −
5	18	0.17 (3) −	0.06 (1) −	0	0.23 (4) −
WF (mg·mL^−1^)					
1.25	10	0.30 (3) −	0.10 (1) −	0	0.40 (4) −
2.5	28	0.32 (9) −	0	0	0.32 (9) −
5	24	0.25 (6) −	0.04 (1) −	0	0.29 (7) −
RO (mM)					
0.35	30	0.17 (5) −	0	0	0.17 (5) −
0.7	40	0.35 (14) −	0.08 (3) −	0.03 (1) −	0.45 (18) −
1.39	22	0.14 (3) −	0.14 (3) −	0	0.27 (6) −
2.78	52	0.21 (11) −	0	0.04 (2) −	0.25 (13) −
SY (mM)					
0.16	22	0.23 (5) −	0	0	0.23 (5) −
0.32	10	0.30 (3) −	0	0	0.30 (3) −
0.63	32	0.28 (9) −	0	0	0.28 (9) −
1.26	22	0.32 (7) −	0	0	0.32 (7) −
SI (mM)					
0.15	12	0.42 (5) −	0	0	0.42 (5) −
0.29	8	0.25 (2) −	0	0	0.25 (2) −
0.58	22	0.27 (6) −	0.09 (2) −	0.05 (1) −	0.41 (9) −
1.16	28	0.25 (7) −	0.04 (1) −	0	0.29 (8) −
RO + SY + SI (mM)					
*a* ^2^	38	0.29 (11) −	0	0	0.29 (11) −
*b*	26	0.27 (7) −	0.15 (4) +	0	0.42 (11) −
*c*	17	0.18 (3) −	0	0	0.18 (3) −
*d*	12	0.25 (3) −	0.08 (1) −	0	0.33 (4) −

**^1^** Statistical diagnoses: + (positive) and − (negative) [40,41]. Significance levels α = β = 0.05, one-sided test without Bonferroni correction; ^2^ Letters *a*–*d* correspond to the lowest, two intermediate and highest concentrations respectively assayed for each single compound once their mixture is assayed.

#### 3.1.1. Toxicity Assays

Table 1 summarizes the toxicity results obtained for analyzed samples expressed as percentage of emerged adults from treatment compared with the emerged adults from the negative control (survival control corrected).

All treatments at all assayed concentrations significantly affected *D. melanogaster* survival except plant samples of *B. officinalis* BF at concentrations 1.25 and 2.5 mg·mL^−1^ and *B. officinalis* WF at 1.25 mg·mL^−1^. The highest concentration of *B. officinalis* BF reduced the *D. melanogaster* survival to less than 20%. Intermediate and highest *B. officinalis* WF assayed concentrations decreased *D. melanogaster* survival to 63.11% and 71.33% respectively. Regarding borage toxicity, the American Herbal Products Association’s Botanical Safety Handbook recommends *Borago* ssp. leaf consumption sporadically due to their pyrrolizidine alkaloid content [46,47]. However, current revisions of *Borago* ssp. properties suggest that the complex bioactive compound leaf composition of this species is more beneficial than harmful for human health because of its phenolic content [3]. This fact could explain the difference we have found between *B. officinalis* BF and WF toxicity levels. On average, the bioactive compounds reduced *D. melanogaster* larval survival by around 50% (LD_50_), normal values for toxicity assays and no dose effect was observed. RO showed the largest reduction in survival, with the highest RO concentration being the most toxic treatment (21.33%). Other authors have also found RO toxicity by oral administration [48]. However, these authors recommend the use of RO in human inflammatory diseases because of its protective effect in the stomach unlike commonly used anti-inflammatory products that possess serious disadvantages for human health. The addition of H_2_O_2_ to the medium in combined treatments contributed to reducing *D. melanogaster* larval survival in all samples when compared to simple treatments, with the exception of the highest *B. officinalis* BF concentration as well as all RO assayed concentrations and highest mixture concentration. These treatments had a protective effect against H_2_O_2_ damage (detoxification), interfering with H_2_O_2_ oxidative action and slightly increasing *D. melanogaster* larval survival. Nevertheless, only in the case of RO treatments this effect was significant. Contrarily, the application of RO mixed with SY and SI (mixture treatment) did not present any protective additive effects with the exception of highest tested concentration. Thus, the addition of H_2_O_2_ to the medium in mixture treatments reduced *D. melanogaster* survival to a greater degree than applying RO alone in combined treatments. However, the mixture survival ended up quite similar to RO survival in combined treatments (survival average of 42.67 and 41.28 respectively). Previous reports showed that the *B. officinalis* beneficial effect on health depends on the composition of phenolics having synergic effects [20,49]. This fact could explain why the mixture of selected bioactive compounds did not exert the same protective effects as RO when it is added alone to a larvae feeding medium in combined treatments. *B. officinalis* WF treatments resulted in the highest survival reduction (average of 66.32%) when combining with H_2_O_2_. Moreover, the combined (H_2_O_2_) treatment at the highest *B. officinalis* WF concentration produced the highest reduction of *D. melanogaster* survival decreasing this value to 17.33%. The H_2_O_2_ toxic effect was enhanced also by lowest and intermediate *B. officinalis* BF concentrations with an average survival reduction of ~50%.

#### 3.1.2. Genotoxicity Assays

Table 2 summarizes the genotoxicity results obtained in the Somatic Mutation and Recombination Test (SMART) as total mutations per wing observed in treatments with *B. officinalis* plant and bioactive compound samples.

It is remarkable that no concentration of plant samples was significantly different from the negative control, but contrarily, some of the treatments showed lower mutation rates (from 0.20 to 0.30) than the negative control (0.32). Although a healthy and non-genotoxic effect of many herbal products is generally expected, it is necessary to empirically check this assumption for parts of the plants that are usually consumed [1,50]. This result is also displayed for plant phenolic products for which pharmacological potential has been widely tested but no complete understanding of their mechanism of action has been elucidated [51].

At present, very little is known about the lack of genotoxicity of *B. officinalis* plants with no direct work reporting genotoxic effects, although a previous work determined the genotoxicity of pirrolizidine alkaloids (compounds present in *B. officinalis* plants) using SMART [52]. This work classified pirrolizidine compounds as genotoxic but this effect varied widely depending on their chemical structures. Similarly to the plant sample results, the main bioactive constituents of *B. officinalis*, RO, SY and SI phenols, were not mutagenic in the *Drosophila* wing spot test as expected from the negative results for the plant. A multitude of beneficial biological activities have been described for RO (astringent, antioxidative, anti-inflammatory, antimutagen, antibacterial and antiviral), so the non-mutagenic results obtained in the wing spot test were expected [24]. Our results also agree with those of Pereira *et al.* [53] that showed no genotoxic effect of RO (doses of 2 and 8 mg·kg^−1^) using the comet assay in brain tissue and peripheral blood in rats. In conclusion, *B. officinalis* plants and their selected components did not exert any DNA damage on the *mwh/flr* eukaryotic system of *D. melanogaster*.

#### 3.1.3. Antigenotoxicity Assays

In this work we present results on the antigenotoxic activity of *B. officinalis* leaves and petioles which could be considered as a health benefits index. Our results for combined treatments in the SMART, showed in Table 3, account for the desmutagenic activity of the selected substances when assayed against H_2_O_2_.

**Figure 1:**
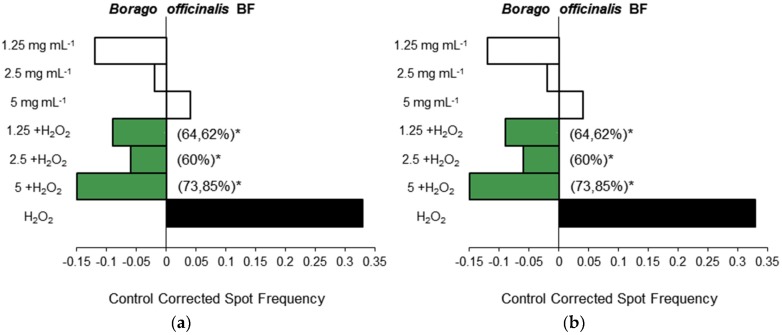
Antigenotoxic activity of *Borago officinalis* plant material: (**a**) blue flowered (BF) and (**b**) white flowered (WF) plant material expressed as mutation frequency corrected to control. Strength of inhibition on the capability of H_2_O_2_ (0.12 M) to induce mutated cells is also shown (Inhibition Percentage in brackets). White columns correspond with tested concentrations of simple treatments, green with combined treatments and black with spot frequencies induced by H_2_O_2_. ^*^ Significance levels with respect to the positive control (H_2_O_2_) group (*p* ≤ 0.05).

**Figure 2:**
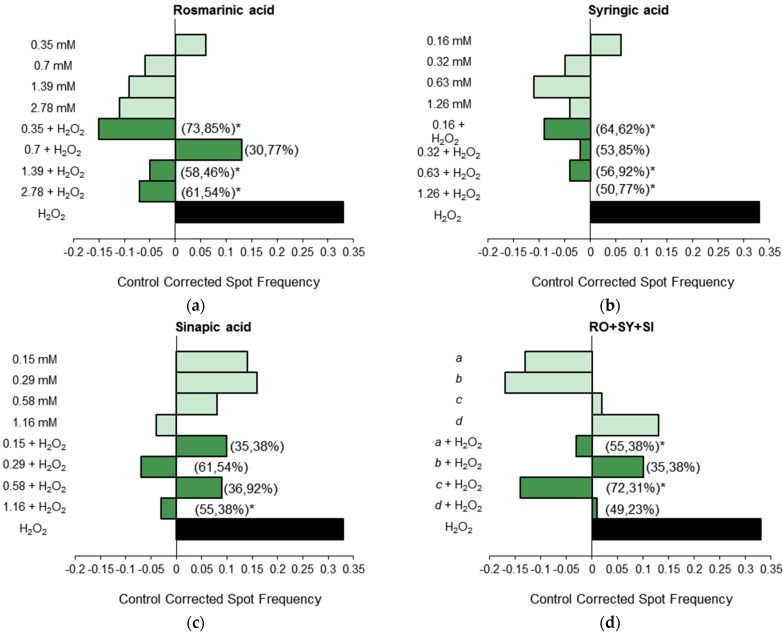
Antigenotoxic activity of *Borago officinalis* bioactive compounds: (**a**) RO; (**b**) SY; (**c**) SI and (**d**) mixture (RO + SY + SI) expressed as mutation frequency corrected to control. Strength of inhibition on the capability of H_2_O_2_ (0.12 M) to induce mutated cells is also shown (Inhibition Percentage in brackets). Light green columns correspond with tested concentrations of simple treatments, green with combined treatments and black column corresponds to spot frequencies induced by H_2_O_2_. Letters *a*–*d* in graphic (**d**) correspond to the lowest, two intermediate and highest concentrations respectively assayed for each single compound once their mixture is assayed. * Significance levels with respect to the positive control (H_2_O_2_) group (*p* ≤ 0.05).

The inhibition percentage (IP) ranged between 30.77% and 73.85% in tested samples. The highest detoxification potential appeared in the highest *B. officinalis* BF concentration (Figure 1a) as well as RO at 0.35 mM (Figure 2a). The lowest detoxification potential corresponded to RO treatments at 0.7 mM (Figure 2a). All these samples corresponded to combined treatments (adding H_2_O_2_ to samples). No dose effect relationship was observed. The detoxifying ability of highest *B. officinalis* BF and lowest RO assayed concentrations against mutations produced by H_2_O_2_ can be explained by the direct interaction of phenols contained in the plants which act as scavengers of reactive oxygen species before the larvae uptake and H_2_O_2_ reaches the DNA [54]. In this respect, RO, SY and SI behaved as desmutagens with a high antioxidative capacity, which has also been shown when they are extracted from borage defatted seeds [55].

Our results for RO bioactive compound are in accordance with prior reports showing its protective effect against H_2_O_2_ damage in other *in vivo* systems like rats as well as *in vitro* systems [56,57]. This antigenotoxic effect has also been demonstrated against the DNA damage brought on by the mutagen ethyl methanesulfonate in males from *D. melanogaster* using the sex-linked recessive lethal (SLRL) test [58]. The other phenolics assayed, SY and SI, possess lower antigenotoxic activity with SI being the least effective in reducing mutations induced by H_2_O_2_ (Figure 2b,c). In accordance with these results, SI has been recently used in order to determine its genotoxic/antigenotoxic activity in the V79 cell line [59]. This phenolic was found to be antigenotoxic but in a way that depends on the dose, with the lower concentrations (below 2 mM, as our assayed concentrations) being those that significantly reduce DNA damage. As discussed for toxicity results, no additive effect in phenolic mixture was found in any assayed concentration (Figure 2d). In this sense, phenolic borage content varies depending on the plant stage, tested phenolics being the major bioactive constituents during plant growth [20,49]. This fact might suggest that the antigenotoxic effect found in our samples corresponds to a specific phenolic or the addition of each phenolic effect. However, our results showed that phenolic effects are not additives but synergic.

### 3.2. In Vitro Assays

#### Cytotoxicity Assays

**Figure 3:**
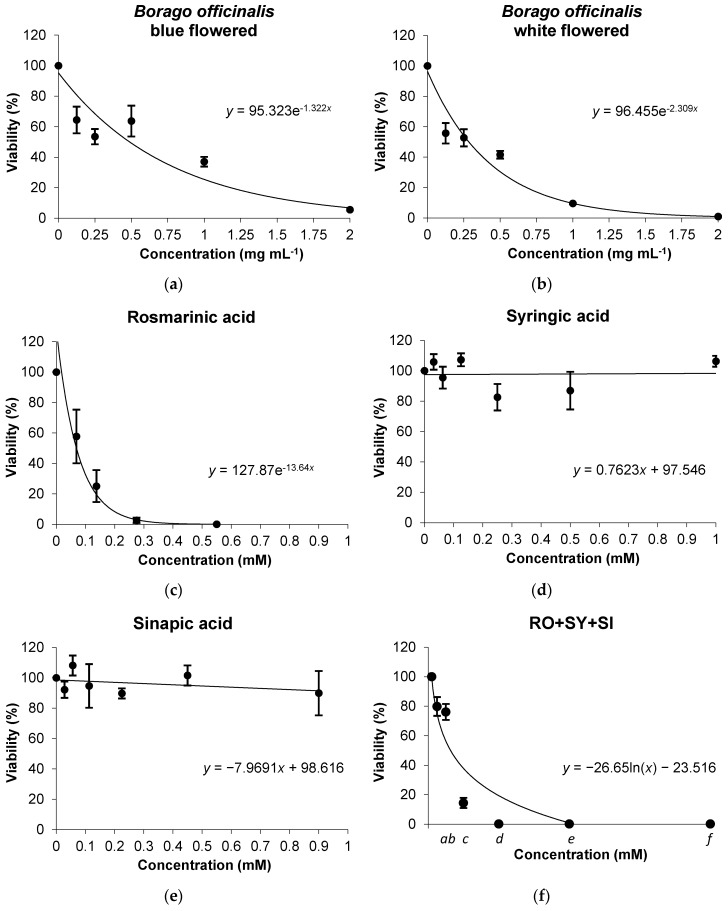
Survival of HL-60 cultures treated with different concentrations of: *Borago officinalis* (**a**) blue flowered (BF) and (**b**) white flowered (WF) plant material; and bioactive compounds: (**c**) RO; (**d**) SY; (**e**) SI and (**f**) mixture (RO + SY + SI; italic letters from *a*–*f* correspond to the concentrations respectively assayed for each single compound once their mixture is assayed.). Survival estimated regressions are plotted as percentages with respect to the control counted from at least three independent experiments (mean ± SD).

The human acute promyelocytic leukemia cell line HL-60 has been used as a model on a wide variety of substances that are candidates to be used as anticarcinogens and has proved to be a robust test system for pilot screening experiments [30,39,60]. That is why we have selected this system to elucidate the inhibitory capacity of tumour growth for the different samples studied. Our results are shown in Figure 3 as the relative HL-60 growth rate with different concentrations of *B. officinalis* BF and WF plant samples and their main active components (RO, SY and SI) regarding their concurrent control cultures.

A dose-response curve was observed for *B. officinalis* BF and WF plant material (Figure 3a,b) which exhibited IC_50_ values of 0.49 and 0.28 mg·mL^−1^ respectively. This cytotoxic effect of borage was also found in the Vero line of African green monkey kidney cells with an IC_50_ value of 0.2 mg·mL^−1^ (similar to that obtained for our borage WF samples) [61].

In the case of phenolic compounds, the IC_50_ could only be determined for RO (0.07 mM) and mixture (0.04 mM of RO equivalent units) samples with a marked slope in the case of phenol mixture. Interestingly, no viable cells could be detected when RO was added to the cell medium (alone or in the mixture) at concentrations over 0.55 mM (Figure 3c,f). Other studied cancer cell lines have been shown to be more sensitive to RO exposure than HL-60 cells, a fact that enhances the disease prevention properties of RO [62,63]. Also, *in vivo* studies in mice conclude that the RO anticarcinogenic activity is related to the activity of this phenol in inhibiting inflammation and scavenging reactive oxygen species [64]. In our experiments, the phenols SY and SI did not affect HL-60 growth (Figure 3d,e). The lack of cytotoxicity in HL-60 experiments that we have found for SY is in accordance with previous determination that indicated no cytotoxic effect of SY in extracts of *Elaphomyces granulates* at concentrations up to ~31 µg·mL^−1^ using this cell line [65]. Moreover, Fabiani *et al.* [66] found that SY did not induce apoptosis in HL-60 cell when is applied to the cell medium at a concentration of 0.1 mM. It has been reported that SI biotransformation by plant peroxidases results in an anticarcinogenic effect from its derivates in HL-60 cells [67]. This fact could explain the difference that we found between its antigenotoxic effect in *D. melanogaster* individuals and the lack of SI cytotoxicity in HL-60 cells, SI derivates being responsible for healthy actions instead of the phenol. As in the case of antigenotoxicity experiments, the cytotoxic effect of the mixture did not correspond to the addition of each individually assayed phenolic. Moreover, the fact that mixture samples presented the highest anticarcinogenic effect proves that the phenolic mixture produces a synergic healthy effect.

## 4. Conclusions

We have provided a primer on antigenotoxicity and tumoricide activities of edible parts (leaves and petioles) of two borage varieties and some of its bioactive principles. The *in vivo* assays showed their safe use for human consumption and their antigenotoxicity potency, supporting their protective DNA damage activity and consequently their health benefits. Our results in the *in vitro* assays highlight *B. officinalis* fresh plant use as a nutraceutical plant and as a potential source of dietary bioactives with an outstanding anticarcinogenic activity. In this sense, *B. officinalis* is a desirable Mediterranean plant adapted to the European climate and a good source of pharmaceutical products, which has made *B. officinalis* a fashionable topic in plant research. Borage breeders have to take this eventual insight as a unique opportunity. Exploitation of this vegetable could be focused on a dual perspective: on the one hand, these cultivars could be partially used for bioactive resources and on the other, as a part for growing a unique plant. The wide spread of *B. officinalis* cultivars for industrial purposes should be used to advise world markets about the pleiotropic uses of this vegetable, not only as a source of products but also as a nutraceutical fresh-consumed plant.

In brief, the varieties studied here show that *B. officinalis* could be put on the table not as a silent partner with other vegetables but as something more than a salad due to its protective and chemopreventive activities.

## Abbreviations

BF: *Borago officinalis* blue-flowered
CO_2_: carbon dioxide
DNA: deoxyribonucleic acid
*flr*: flare
H_2_O_2_: hydrogen peroxide
HL-60: human acute promyelocytic leukemia cell line
IC_50_: half maximal inhibitory concentration
IP: inhibition percentage
LD_50_: median lethal dose
*mwh*: multiple wing-hair
RO: rosmarinic acid
SI: sinapic acid
SMART: somatic mutation and recombination test
SY: syringic acid
T: toxicity
WF: *Borago officinalis* white-flowered
