# Correction: Lozano-Baena, M.-D.; *et al.* Cancer Prevention and Health Benefices of Traditionally Consumed *Borago officinalis* Plants. *Nutrients* 2016, *8*(1), 48

**DOI:** 10.3390/nu8020105

**Published:** 2016-02-19

**Authors:** Nutrients Editorial Office

**Affiliations:** MDPI AG, Klybeckstrasse 64, CH-4057 Basel, Switzerland

Due to mistake during the conversion process, the Figure 1a,b in the original published version were the same [1]. The *Nutrients* Editorial Office wishes to make the following correction to this paper. The correct Figure 1 is shown below:

We would like to apologize for any inconvenience caused to the readers by this mistake.

## Figures and Tables

**Figure 1:**
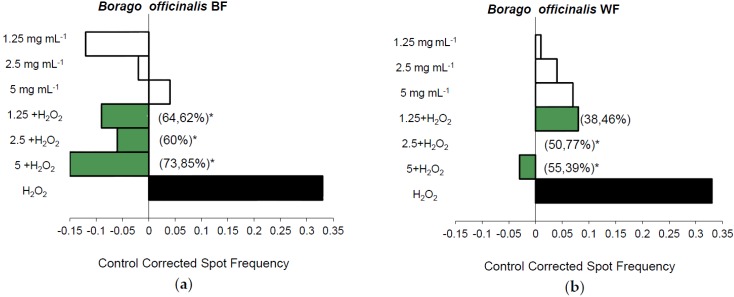
Antigenotoxic activity of Borago officinalis plant material: (**a**) blue flowered (BF) and (**b**) white flowered (WF) plant material expressed as mutation frequency corrected to control. Strength of inhibition on the capability of H_2_O_2_ (0.12 M) to induce mutated cells is also shown (Inhibition Percentage in brackets). White columns correspond with tested concentrations of simple treatments, green with combined treatments and black with spot frequencies induced by H_2_O_2_. * Significance levels with respect to the positive control (H_2_O_2_) group (*p* ≤ 0.05).
